# Predictive model for a second hip fracture occurrence using natural language processing and machine learning on electronic health records

**DOI:** 10.1038/s41598-023-50762-5

**Published:** 2024-01-04

**Authors:** Ricardo Larrainzar-Garijo, Esther Fernández-Tormos, Carlos Alberto Collado-Escudero, María Alcantud Ibáñez, Fernando Oñorbe-San Francisco, Judith Marin-Corral, David Casadevall, David Donaire-Gonzalez, Luisa Martínez-Sanchez, Lucia Cabal-Hierro, Diego Benavent, Fátima Brañas

**Affiliations:** 1 Orthopedic and Trauma Department, Hospital Universitario Infanta Leonor, Medical School, Universidad Complutense, Madrid, Spain; 2 Orthopedic and Trauma Department, Hospital Universitario Infanta Leonor, Madrid, Spain; 3 Geriatric Department, Hospital Universitario Infanta Leonor, Medical School, Universidad Complutense, Madrid, Spain; 4 Savana Research Group: Medsavana & Savana Research S.L., Madrid, Spain

**Keywords:** Risk factors, Osteoimmunology, Trauma

## Abstract

Hip fractures (HFx) are associated with a higher morbidity and mortality rates, leading to a significant reduction in life quality and in limitation of patient´s mobility. The present study aimed to obtain real-world evidence on the clinical characteristics of patients with an initial and a second hip fracture (HFx) and develop a predictive model for second HFx using artificial intelligence. Electronic health records from one hospital centre in Spain from January 2011 to December 2019 were analysed using *EHRead®* technology, based on natural language processing and machine learning. A total of 1,960 patients with HFx were finally included during the study period after meeting all inclusion and exclusion criteria. From this total, 1835 (93.6%) patients were included in the HFx subgroup, while 124 (6.4%) were admitted to the second HFx (2HFx) subgroup. The mean age of the participants was 84 years and 75.5% were female. Most of comorbidities were more frequently identified in the HFx group, including hypertension (72.0% vs. 67.2%), cognitive impairment (33.0% vs. 31.2%), diabetes mellitus (28.7% vs. 24.8%), heart failure (27.6% vs. 22.4%) and chronic kidney disease (26.9% vs. 16.0%). Based on clinical criteria, 26 features were selected as potential prediction factors. From there, 16 demographics and clinical characteristics such as comorbidities, medications, measures of disabilities for ambulation and type of refracture were selected for development of a competitive risk model. Specifically, those predictors with different associated risk ratios, sorted from higher to lower risk relevance were visual deficit, malnutrition, walking assistance, hypothyroidism, female sex, osteoporosis treatment, pertrochanteric fracture, dementia, age at index, osteoporosis, renal failure, stroke, COPD, heart disease, anaemia, and asthma. This model showed good performance (dependent AUC: 0.69; apparent performance: 0.75) and could help the identification of patients with higher risk of developing a second HFx, allowing preventive measures. This study expands the current available information of HFx patients in Spain and identifies factors that exhibit potential in predicting a second HFx among older patients.

## Introduction

Human beings, especially in developed countries, are continuously improving their longevity and quality of life. This is unfortunately associated with an increase in age-related issues such as hip fractures (HFx), which constitute a substantial socioeconomic burden on society^1,2^. Therefore, it is imperative to assess how factors associated with HFx overall influence elderly health^3^. Falls, a significant concern in this demographic group, often result from a combination of factors such as sarcopenia, cognitive impairments, and poly-pharmacotherapy. Consequently, the management of fall risks, necessitates comprehensive strategies that not only identify these risk factors but also incorporate effective multi-component interventions. This includes the utilization of rehabilitation techniques, which have been shown to significantly reduce the risk of falls and improve cognitive and physical functions in the elderly^4,5^.

Studies indicate that after an initial HFx, approximately 2–10% of patients may experience a second fracture^6^. This new HFx seriously affects patients' quality of life, increasing their chances of becoming dependent on others, significantly negatively impacting their quality of life, and limiting patient autonomy and self-care. Furthermore, a second HFx is associated with increased mortality rates^7^ and a high economic burden, encompassing direct and indirect expenses^8,9^. Although guidelines have been developed to establish anti-osteoporotic drug strategies to prevent a second HFx event, not all patients with a first fracture have the same risk of experiencing a second one. Thus, it is vital to identify the risk factors specifically associated with a second HFx, which will allow for developing selective multimodal strategies to avoid recurrence in at-risk patients.

Identifying risk factors associated with a second HFx is particularly challenging because the target population has several confounders owing to its age and high comorbidity. In fact, second HFx risk factors tend to be empirically associated with risk factors for postmenopausal osteoporosis^10^. However, whether first and second fragility fracture risk factors are similar or whether new factors may exist is unclear. Hence, individualized preventive strategies can only be developed if specific risk factors for a second HFx are identified. The use of artificial intelligence in combination with machine learning (ML) and natural language processing (NLP) techniques on electronic health records (EHRs) provides a new model of population research that can provide further insights on previously unknown aspects related to associations and risk factors for a second HFx in the elderly population.

In the present real-world data (RWD) study, our objective was to characterize and compare patients with HFx who presented with a second HFx and to develop a predictive model for a second HFx. To this end, we proposed the use of EHRead®, a technology that applies NLP and ML, to analyse the free-text information on EHR, as well as statistical techniques to compare these patients and to develop a competing risk predictive model for a second HFx^11^. Our findings will help to define the target population for using multimodal treatment strategies to minimize the occurrence of a second HFx in at-risk patients.

## Methods

### Study design and population

**Figure 1:**
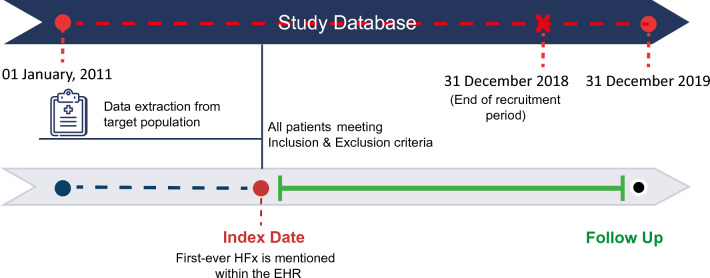
Study design. Data were extracted from EHRs corresponding to the study period (from January 1st, 2011 to December 31st, 2019) and were analysed using EHRead® technology. The full analysis set (i.e., all HFx patients who fulfil all inclusion/exclusion criteria) was comprised of 1836 patients.

A retrospective cohort study was obtained using RWD collected at Hospital Universitario Infanta Leonor in Madrid between January 2011 and December 2019 (Fig. 1). Patients aged 65 years or older who suffered HFx during the study period (SP) were included. Index or inclusion date (ID) was defined as the first time within the SP when HFx was mentioned. Patients were considered to have a second HFx if a contralateral HFx was detected during the SP. Patients were excluded if fracture laterality was not detected, had less than one-month follow-up (FU), if a contralateral HFx was detected during the first month after the first HFx, and if a previous HFx existed before the ID. Descriptive analyses were stratified by the occurrence or not of a second HFx (2HFx vs. HFx). All patients were followed until the second HFx, death, their last report, or the end of SP, whichever came first. All available emergency, inpatient, outpatient records and hospital pharmacy and laboratory data were used to characterize patients and events of interest at baseline and during FU.

### Study variables

Conceptual definitions of all study variables were pre-specified and mapped to clinical entities present in SNOMED clinical terms (CT) using the SNOMED CT browser. SNOMED CT is a systematically organized computer-processable collection of medical terms used in clinical documentation^12^. The clinical accuracy of the conceptual definitions and entity mapping was reviewed and approved by two specialists, an orthopaedic surgeon, and a rheumatologist. Mapped clinical entities were then extracted from EHRs using EHRead®, a proprietary technology that uses NLP and ML to extract clinical entities and their context from free text^13^. To ensure the quality of data extraction, EHRead® performance was externally evaluated on a set of 11 key clinical entities (including those used to identify the target population and its most significant characteristics). Performance evaluation was conducted by evaluating EHRead® results in a corpus of medical records in which key entities were annotated by specialists from the participating institution^11^. Performance results are provided in Suppl. Table 1.

After clinical entity extraction, variables were constructed by applying dedicated data wrangling operations to their mapped entities, leveraging specific NLP parameters generated by dedicated ML models (e.g., negation, temporality, attributes, etc.) and record-specific metadata (e.g., date, medical department, record type, etc.). Socio-demographic characteristics included age, sex, tobacco, and alcohol use. Clinical characteristics included comorbidities, haematology and biochemistry parameters, use of cardiovascular or neurological medication, and measures of disability for ambulation such as unsteady gait, use of walking assistance, visual problems, and the Barthel Index. For baseline characterization regarding pharmacological treatments and laboratory parameters, we used records from the 6 months before ID. For detecting a second HFx, EHRs from the FU period were also used (from the first-time HFx was mentioned until the last EHR available within the SP).

### Descriptive analysis (HFx vs 2HFx)

Baseline and FU characteristics were described separately for HFx and 2HFx patients. In addition, an ad hoc descriptive analysis of mortality corresponding to the median time until refracture (1.3 years) was performed in the HFx group to better understand the potential influence of death as a competitive event. The length of the follow-up time for model development was determined considering the competing event distribution across time. Frequency tables were used to describe categorical variables. The distribution of continuous variables was summarized using the mean, standard deviation (SD), median, and range (Q1, Q3). For boolean variables such as comorbidities or symptoms, which are assumed to be reported if present in patients, their absence in EHRs was imputed as a true absence (i.e., the patient lacked the comorbidity/symptom). For multi-level (≥ 3) categorical variables such as lifestyle factors (e.g., toxic habits) and numerical variables, their absence was not imputed, and their missing rates are reported.

### Predictive model for 2HFx

A subdistribution hazard (Fine-Gray) competing-risk model for the occurrence of 2HFx was developed, with death as the competitive event, under R version 4.1.1. using the ‘riskRegression’ package. The necessary steps for predictive model development are described below:

#### Population selection and characteristics

To ensure appropriate FU, given that the median time to refracture for 2HFx patients was 1.3 years- as shown in the descriptive analyses, patients with ID less than one year before the end of SP were excluded (Fig. 1). A detailed descriptive analysis of this group of patients was conducted to better understand the potential predictors of 2HFx and death. The descriptive analyses were stratified based on event occurrence, with patients categorized as either event-free, having experienced a primary event, or having experienced a competing event. The median FU from all patients (4.6 years) was used to determine the most appropriate time point for capturing the events of interest to predict (five years). The cause-specific cumulative incidence function was used to describe the absolute risk of experiencing either a primary or competing event over time.

#### Variable selection

The maximum number of predictors to be included in the model was defined based on the sample size and observed event rate, as described by Riley et al.^14^. This method seeks to minimize the overfitting risk by limiting the number of predictor parameters used to train a clinical prediction model. Considering this, a limited list of predictors was selected following clinical criteria based on their known (or potential) association with the outcome. Variables with more than 10% missing data were not considered for model training, except for smoking habits, in which patients with unknown smoking status were assumed to be non-smokers. Once the initial set of predictors was obtained, backward feature selection was applied to determine the minimum number of predictors needed without compromising model performance. Using this procedure, predictor variables were sequentially removed based on their multivariable *p*-value (removing variables with the highest value in each iteration) until a significant drop in predictive performance was detected. Specific predictors were forced into the model due to their clinical importance. Model discrimination (c-index, the area under the curve [AUC]) was calculated from 200 bootstrap samples of the whole study population to assess model performance in each iteration. Box plots of the performance measures from all bootstrapped samples of each iteration were produced for each step. To objectively determine the most suitable cut-off point, performance results between each iteration were statistically compared using the Wilcoxon test until a significant drop in performance was observed.

#### Internal model validation

The final model was obtained and internally validated using bootstrapping, allowing for optimism correction of model performance. Model development was conducted as follows: firstly, the apparent performance (c-index and AUC) was obtained by estimating the model on the full population (i.e., the whole study population served as training and validation set). Secondly, to estimate the expected performance drop in external datasets due to potential overfitting, models were re-estimated and their apparent performance was obtained in 200 bootstrap samples from the original population. Model optimism was then obtained by testing each of the bootstrap models on the full, original population, and then subtracting the performance obtained in each test from the apparent performance of the corresponding bootstrap model, as previously described^15^. Finally, we obtained the optimism-corrected performance by adjusting the initial apparent performance metrics with the averaged optimism metrics.

### Ethics

To ensure patient confidentiality, all information was collected from the pseudonymized EHR, from which only study-specific variables were extracted. The collected variables were then aggregated in a study database that was anonymized for the analysis stage. The study was approved by the **Independent Ethics Committee of Hospital Universitario Infanta Leonor** and adhered to legal and regulatory standards for good research practices outlined in the most recent edition of the Helsinki Declaration. The patient agreement was not required as all information was gathered from an anonymous EHR.

## Results

### Descriptive analyses (HFx vs. 2HFx)

**Figure 2:**
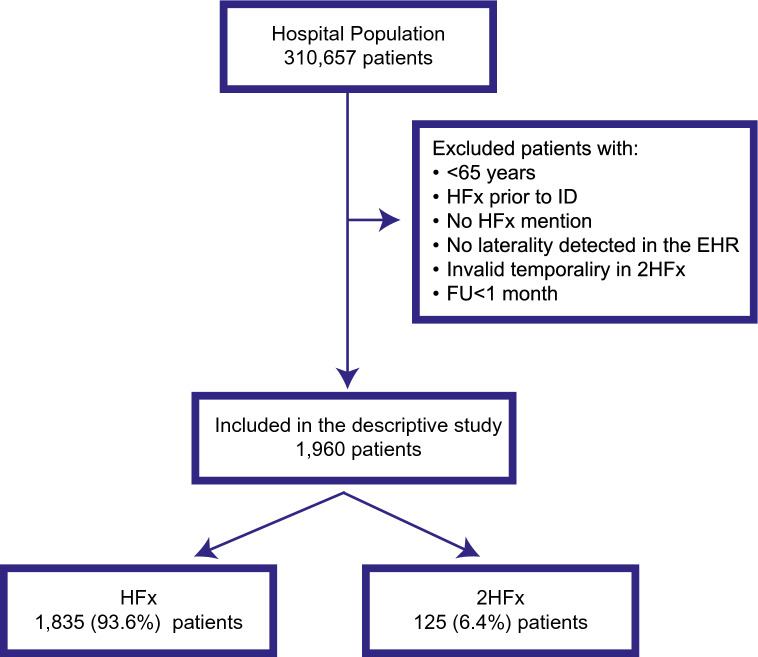
Workflow of the study population EHR: Electronic health records; FAS: Full analysis set; HF: Hip fracture.

From a hospital population of 310,657 patients, a total of 1960 patients were finally included during the SP after meeting all inclusion and exclusion criteria. In this group, 1835 (93.6%) patients were included in the HFx subgroup, while 124 (6.4%) were admitted to the 2HFx subgroup (Fig. 2).

The patient's demographic and clinical characteristics at baseline, overall, and by study subgroup are presented in Table 1. The mean age of the participants was 84 years (SD 7 years), and 75.5% were female (75.2% in HFx patients and 80% in 2HFx patients). Alcohol consumption and smoking habits were detected in 21.9% and 12.7% of patients, respectively. Cardiovascular risk factors were comorbidities more frequently observed (88.1%), followed by anaemia (71.8%), osteoporosis (40.1%), and cognitive impairment (32.9%). Most of the comorbidities were more frequently described in the HFx patients, such as hypertension (72.0% vs. 67.2%), cognitive impairment (33.0% vs. 31.2%), diabetes mellitus (28.7% vs. 24.8%), heart failure (27.6% vs. 22.4%) and chronic kidney disease (26.9% vs. 16.0%). Baseline laboratory values are shown in Suppl. Table 2. It shows that, in general, the hemogram and biochemistry values were similar between both groups. The overall in-hospital mortality rates were 19.0% (n = 372), 19.3% in HFx patients (n = 354), and 14.4% in 2HFx patients (n = 18). The timing between initial HFx and death yielded a median (Q1–Q3) of 201.50 (33.25, 732.00) days in the HFx patients and 682.50 (464.50, 1027.75) days in 2HFx patients. The overall median FU was 4.6 years.

**Table 1:** Demographic and clinical characteristics at baseline.

	HFx (n = 1835)	2HFx (n = 125)	Overall (n = 1960)
Demographics			
Gender, n (%)			
N	1835 (100)	125 (100)	1960 (100)
Female	1380 (75.2%)	100 (80.0%)	1480 (75.5%)
Male	455 (24.8%)	25 (20.0%)	480 (24.5%)
Age at index, years			
N, n (%)	1835 (100)	125 (100)	1960 (100)
Mean (SD)	83.88 (6.99)	82.77 (6.31)	83.81 (6.95)
Median (Q1-Q3)	85.00 (79.00, 89.00)	84.00 (79.00, 87.00)	85.00 (79.00, 89.00)
(min, max)	(65,102)	(66, 95)	(65, 102)
Toxic habits			
Alcohol, n (%)			
Missing	1318 (71.8%)	83 (66.4%)	1401 (71.5%)
Consumer	395 (21.5%)	34 (27.2%)	429 (21.9%)
No consumer	122 (6.6%)	8 (6.4%)	130 (6.6%)
Tobacco, n (%)			
Missing	1556 (84.8%)	111 (88.8%)	1667 (85.1%)
Smoker	237 (12.9%)	11 (8.8%)	248 (12.7%)
No smoker	42 (2.3%)	3 (2.4%)	45 (2.3%)
Comorbidities			
Cardiovascular risk factors, n (%)	1619 (88.2%)	108 (86.4%)	1727 (88.1%)
Dyslipidemia, n (%)	1587 (86.5%)	107 (85.6%)	1694 (86.4%)
Anaemia, n (%)	1320 (71.9%)	88 (70.4%)	1408 (71.8%)
Hypertension, n (%)	1321 (72.0%)	84 (67.2%)	1405 (71.7%)
Osteoporosis, n (%)	736 (40.1%)	50 (40.0%)	786 (40.1%)
Cognitive impairment, n (%)	606 (33.0%)	39 (31.2%)	645 (32.9%)
Diabetes mellitus, n (%)	527 (28.7%)	31 (24.8%)	558 (28.5%)
Heart failure, n (%)	506 (27.6%)	28 (22.4%)	534 (27.2%)
Chronic Kidney disease, n (%)	493 (26.9%)	20 (16.0%)	513 (26.2%)
Hyperparathyroidism, n (%)	326 (17.8%)	20 (16.0%)	346 (17.7%)
Dementia, n (%)	370 (20.2%)	16 (12.8%)	386 (19.7%)
Anxiety, n (%)	254 (13.8%)	22 (17.6%)	276 (14.1%)
COPD, n (%)	258 (14.1%)	14 (11.2%)	272 (13.9%)
Rheumatoid arthritis, n (%)	217 (11.8%)	14 (11.2%)	231 (11.8%)
Ischemic cardiopathy, n (%)	198 (10.8%)	12 (9.6%)	210 (10.7%)
Stroke, n (%)	181 (9.9%)	8 (6.4%)	189 (9.6%)
Hip prosthesis, n (%)	178 (9.7%)	6 (4.8%)	184 (9.4%)
Hypothyroidism, n (%)	159 (8.7%)	10 (8.0%)	169 (8.6%)
Osteopenia, n (%)	142 (7.7%)	10 (8.0%)	152 (7.8%)
Parkinson disease, n (%)	75 (4.1%)	6 (4.8%)	81 (4.1%)
Asthma, n (%)	63 (3.4%)	5 (4.0%)	68 (3.5%)
Gout, n (%)	61 (3.3%)	6 (4.8%)	67 (3.4%)
Hyperthyroidism, n (%)	49 (2.7%)	3 (2.4%)	52 (2.7%)
Hyperuricemia, n (%)	42 (2.3%)	4 (3.2%)	46 (2.3%)
Knee implant, n (%)	35 (1.9%)	1 (0.8%)	36 (1.8%)
Dyspepsia, n (%)	28 (1.5%)	1 (0.8%)	29 (1.5%)
Transient ischemic attack, n (%)	4 (0.2%)	1 (0.8%)	5 (0.3%)

*COPD* Chronic obstructive pulmonary disease; *HFx* Hip fracture; *2HFx* Second hip fracture.

The ad hoc descriptive analysis based on refracture vs. mortality rates across time is shown in Suppl. Table 3. The median time until refracture was 1.3 years; thus, a maximum follow-up period of 2 years after the first fracture was allowed. Within the HFx subgroup of patients, 1605 of them (87.5%) survived within the follow-up period, while 230 patients (12.5%) died within the same time interval. There were substantial differences in comorbidities observed between these two subgroups: patients who died had a higher prevalence of most of them, especially cardiovascular risk factors (95.2% and 87.2%), chronic kidney disease (53.0% and 23.1%), heart failure (52.2% and 24.0%), and stroke (22.6% and 8.0%) compared to patients who survived.

### Predictive model for 2HFx

**Figure 3:**
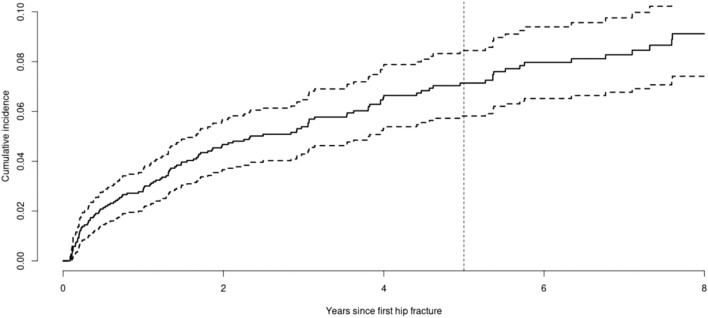
Cumulative incidence curve for 2HFx at 8 years after ID (initial HFx). The graph depicts the cumulative incidence curve for a 2HFx over an eight-year period following the first hip fracture. Dashed vertical line represents the median FU for all patients, while dashed horizontal lines represent 95% Confidence Intervals. Dashed vertical line represents the period for which the model was developed.

After excluding patients without the required follow-up, a total of 1730 patients were included in the model development, 1301 (75.2%) in the event-free group (w/o 2HFx), 109 (6.3%) in the primary event group (2HFx), and 320 (18.5%) in the competing event group (HFx patients who died before having time for a re-fracture). Based on the median FU from all patients (4.6 years), five years was determined to be the most appropriate time point for assessing the risk of 2HFx. The cumulative incidence function for the study period is presented in Fig. 3, highlighting the five years FU timepoint selected for model development.

A total of 26 features were initially selected as potential predictors based on the clinical criteria. Table 2 presents the descriptive results at the baseline across the three subgroups. Mean age was higher in the competing event subgroup (85.4 years) than in the w/o 2HFx (83.3 years) or 2HFx (82.7 years) subgroups, while the proportion of females was lower (61.6%, 78.1%, and 80.7% respectively). Notably, patients who experienced the competing event had higher proportions of several medical conditions than the other groups, such as heart disease (54.4%, 32.2%, and 25.7%, respectively), renal failure (49.1%, 24.7%, and 21.1%, respectively), and stroke (26.6%, 10.6%, and 9.2%, respectively).

**Table 2:** Descriptive analysis for all the potential predictors at baseline in event-free patients (without 2HFx), in patients who suffered a primary event (2HFx) or a competing event (death) in the 5 years period.

	Event-free (w/o 2HFx) N = 1301 (75.2%)	Primary event (2HFx) N = 109 (6.3%)	Competing event (Death) N = 320 (18.5%)	Total N = 1730
Age at index*†				
Mean (SD)	83.28 (6.92)	82.75 (6.48)	85.36 (6.95)	83.63 (6.95)
Median (Q1, Q3)	84.00 (79.00, 88.00)	84.00 (79.00, 87.00)	86.00 (81.00, 90.00)	84.00 (79.00, 89.00)
Female sex*†	1016 (78.1%)	88 (80.7%)	197 (61.6%)	1301 (75.2%)
Osteoporosis*†	500 (38.4%)	39 (35.8%)	114 (35.6%)	653 (37.7%)
Dementia*†	479 (36.8%)	42 (38.5%)	167 (52.2%)	688 (39.8%)
Osteoporosis treatment†	332 (25.5%)	29 (26.6%)	54 (16.9%)	415 (24.0%)
Heart disease†	419 (32.2%)	28 (25.7%)	174 (54.4%)	621 (35.9%)
COPD†	187 (14.4%)	12 (11.0%)	96 (30.0%)	295 (17.1%)
Asthma†	55 (4.2%)	3 (2.8%)	21 (6.6%)	79 (4.6%)
Renal Failure†	321 (24.7%)	23 (21.1%)	157 (49.1%)	501 (29.0%)
Hypothyroidism†	128 (9.8%)	13 (11.9%)	29 (9.1%)	170 (9.8%)
Stroke†	138 (10.6%)	10 (9.2%)	85 (26.6%)	233 (13.5%)
Malnutrition†	133 (10.2%)	14 (12.8%)	41 (12.8%)	188 (10.9%)
Visual deficit†	35 (2.7%)	4 (3.7%)	6 (1.9%)	45 (2.6%)
Anemia†	910 (69.9%)	67 (61.5%)	249 (77.8%)	1226 (70.9%)
Walking assistance†	385 (29.6%)	38 (34.9%)	130 (40.6%)	553 (32.0%)
Pertrochanteric fracture†	435 (33.4%)	38 (34.9%)	104 (32.5%)	577 (33.4%)
Smoking habit	153 (11.8%)	11 (10.1%)	60 (18.8%)	224 (12.9%)
Diabetes mellitus	391 (30.1%)	31 (28.4%)	122 (38.1%)	544 (31.4%)
Hyperthyroidism	46 (3.5%)	3 (2.8%)	15 (4.7%)	64 (3.7%)
Hypertension	986 (75.8%)	77 (70.6%)	267 (83.4%)	1330 (76.9%)
Parkinson disease	64 (4.9%)	6 (5.5%)	17 (5.3%)	87 (5.0%)
Rheumatoid arthritis	216 (16.6%)	20 (18.3%)	63 (19.7%)	299 (17.3%)
Unsteady gait	63 (4.8%)	5 (4.6%)	13 (4.1%)	81 (4.7%)
Central Nervous System medication	687 (52.8%)	56 (51.4%)	194 (60.6%)	937 (54.2%)
Cardiovascular medication	485 (37.3%)	37 (33.9%)	147 (45.9%)	669 (38.7%)
Subcapital fracture	385 (29.6%)	30 (27.5%)	93 (29.1%)	508 (29.4%)

Color code is used for selected predictors indicating if the proportion of patients increase (red) or decrease (green) in primary and competing events in respect with the event-free patients.

*w/o* without.

*Predictors that are forced to be included in the model due to clinical reasons.

^†^Predictors selected by the backward selection for the final model.

For the development of the competitive risk model, from the 26 first assessed features (Table 2), a total of 16 predictors were selected for inclusionin the final model: age at index, female sex, osteoporosis, dementia, osteoporosis treatment, heart disease, COPD, asthma, renal failure, hypothyroidism, stroke, malnutrition, visual deficit, anaemia, walking assistance, and pertrochanteric fracture. Of note, four of them (age at index, female sex, osteoporosis, and dementia) were forced into the models owing to their clinical importance. Suppl. Figure 1 presents the apparent performance of the model according to the number of variables used for the backward selection of predictors.

The final Fine and Gray sub-distribution hazard competing risk model for the second contralateral HFx, including the sub-hazard ratios (sHR) for each predictor, is shown in Table 3. The adjusted model's performance according to the C-index, was 0.58 (apparent performance 0.64) and according to time, the dependent AUC was 0.69 (apparent performance 0. 75).

**Table 3:** Model predictors and coefficients.

	sHR (IC 95%)
Visual deficit	1.82 (0.64–5.19)
Malnutrition	1.63 (0.88–3.01)
Walking assistance	1.46 (0.97–2.20)
Hypothyroidism	1.33 (0.74–2.42)
Female sex	1.25 (0.74–2.10)
Osteoporosis treatment	1.24 (0.78–1.96)
Pertrochanteric fracture	1.22 (0.81–1.82)
Dementia	1.10 (0.73–1.68)
Age at index	0.99 (0.96–1.01)
Osteoporosis	0.97 (0.63–1.48)
Renal Failure	0.82 (0.49–1.36)
Stroke	0.77 (0.40–1.47)
COPD	0.74 (0.37–1.46)
Heart disease	0.70 (0.43–1.15)
Anaemia	0.63 (0.39–1.01)
Asthma	0.62 (0.19–1.99)

## Discussion

The present study aimed to characterize HFx patients who developed or did not a second HFx and perform a predictive model for this recurrence by extracting NLP terms from EHRs. A total of 1960 patients with HFx were included in the study, 6.4% of whom presented with a second HFx. Notably, patients with a second HFx had fewer comorbidities and a lower in-hospital mortality rate than patients with a unique HFx. The predictive model identified 16 predictors of a second HFx, including demographic and clinical characteristics such as comorbidities, medications, measures of disability for ambulation, and type of refracture. Specifically, those predictors with different associated risk ratios, sorted from higher to lower risk relevance, were visual deficit, malnutrition, walking assistance, hypothyroidism, female sex, osteoporosis treatment, pertrochanteric fracture, dementia, age at index, osteoporosis, renal failure, stroke, COPD, heart disease, anaemia, and asthma. The model showed good performance and could help identify patients at a higher risk of developing a second HFx, thereby allowing for preventive measures. Overall, our study shows that NLP methodology can be a powerful tool for identifying variables that exhibit potential in predicting a second HFx among older patients.

Several studies have attempted to identify the risk factors for a second HFx event, including female sex, advanced age, osteoporosis, and comorbidities^16^. Nonetheless, well-established risk factors for postmenopausal osteoporosis and associated HFx do not necessarily apply to patients who undergo a second HFx. In this regard, studies indicate that a second HFx may occur in individuals with little associated morbidity who have enough autonomy to suffer a second fall, although this is not a risk factor for osteoporosis or hip fracture^16–18^. Our study analysed the risk of a second HFx using a time-to-event analysis, in which deaths occurring during FU were considered a competing event. This approach demonstrated that the population who experienced a second HFx had a lower comorbidity and mortality burden than those with a unique HFx without a recurrence. Moreover, our results regarding an ad hoc mortality analysis in patients without a second HFx showed that those who died presented the highest burden. These findings suggest that the higher comorbidity observed in the population who did not present with a second HFx was mainly driven by those patients who died. The use of death as a competing event enabled us to estimate the marginal probability of an event accurately in the presence of competing events. This methodology is important to consider when analysing the risk of a second HFx, as it provides a more comprehensive understanding of the potential outcomes and factors influencing them^19^. This may help further disentangle the specific risk factors associated with a second HFx.

Despite its significance as a critical and clinically relevant event, no adequate predictive tools are currently available to forecast the occurrence of a second HFx. The Fracture Risk Assessment Tool (FRAX) has been widely used to determine the risk of osteoporotic or hip osteoporotic fractures in the general population over a 10-year time window^20^). However, a review conducted on twelve FRAX studies revealed that the average area under the receiver operating characteristics curve (AUROC) for predicting major osteoporotic fractures was 0.65 (*p* = 0.038)^21^. In addition to the demonstrated limitations of FRAX performance characteristics, no validation of FRAX has been carried out for patients who have experienced a second HFx. Crystal Bone, an algorithm that uses ML to predict short-term fracture risk based on longitudinal EHR data, showed to accurately (AUROC = 0.81) predict 1- to 2-year fracture risk for patients over 50 years old^22^. Nonetheless, it has not been validated in a subset of patients who already have HFx, which may have specific characteristics. In this study, we developed a predictive model for the occurrence of a second contralateral HFx. A cumulative incidence curve provided insights into the timing and frequency of the events of interest, allowing for selecting an appropriate FU time for model development.

Our final model identified 16 significant predictors associated with the occurrence of a second HFx event. These include factors with a negative or positive effect on cumulative incidence. Thus age, osteoporosis, and the presence of comorbidities such as heart disease, COPD, asthma, renal failure, stroke, and anaemia negatively affected the cumulative incidence of a second HFx and seemed to be related to an increase in mortality. Indeed, a second hip fracture event might not have occurred because of the patient's deaths. Moreover, many of these factors might be associated with an increase in sedentary behaviour and, thus, related to a decrease in the probability of suffering falls. Interestingly, female sex, dementia, hypothyroidism, malnutrition, osteoporosis treatment, visual deficit, use of walking assistance, and the occurrence of a pertrochanteric fracture positively affected the cumulative incidence (Table 3). Although scarce data are available regarding risk factors for second HFx, some of these predictors have been previously described. In this sense, a previous study reported that older age and both high and low functional status were positively related to a second HFx, while there was no significant association between the second HFx risk and sex, falls, stroke, BMI, dementia, 4-year weight change, or nursing home residence in either the age- and sex-adjusted or multivariate models^23^. Another study found that being female, elderly, living in a nursing home, and having severe to moderate liver disease may be important predictors of a second HFx^24^. According to these previous results, we found that some variables related to functional status, such as osteoporosis treatment, visual deficit, or the use of walking assistance, were related to a higher incidence of second HFx. Furthermore, our model highlighted some unwidely identified predictors for their potential risk for a second HFx, including dementia^23^*.* This finding is particularly noteworthy because patients with dementia are at an increased risk of falls, which could contribute to the development of subsequent HFx. Interestingly, our study identified osteoporosis treatment as a factor with a positive effect on the second HFx incidence, possibly because patients undergoing these therapies already find themselves in a delicate healthy situation prompt to a second HFx. Altogether, our algorithm has potential benefits for both patients and healthcare providers. Moreover, it would allow a more personalized assessment of fracture risk in patients who have already suffered a HFx, which could improve early patient identification, reduce the morbidity and mortality associated with fractures, and minimize the overall burden on patients and the healthcare system burden on patients and the healthcare system overall.

It is important to acknowledge the limitations of our study and be cautious when considering the potential of these predictors individually, as some of them could be biased by the study itself. First, it is worth noting the study's retrospective nature, which may lead to potential selection bias, while using RWD presents intrinsic limitations. For example, some particular outcome measures that are not routinely performed on clinical practice, such as physical performance measures, were not included. Besides, using detection in EHRs can impact model performance and validity if diagnoses are missed, incorrectly recorded, or not detected because of patient dropout. In this regard, in our study we could not include data regarding body mass index, as more than 95% of patients did not present this variable, weight or height, which were not reported in the free text of their EHRs. Similarly, the assessment of toxic habits, should require a cautious approach when drawing conclusions due to the significant number of missing values. However, the use of SNOMED terminology, a comprehensive and standardized system that is regularly updated and maintained, ensures that the detection of terms is maximized. Furthermore, because we incorporated only predefined, specific variables into our analysis, there remains the possibility that relevant features were not captured by our NLP pipeline, thus introducing the potential for unmeasured confounding or missing some potential contributors in variables which were not feasible for inclusion (e.g. all types of cancers or chemotherapy). Additionally, our study was conducted at a single centre, which helped ensure data homogeneity and mandates that our findings can be replicated or enriched in other populations. Despite these limitations, our results highlight the need for more research to develop accurate predictive models for a second HFx, as this could help clinicians identify high-risk patients for preventive interventions, ultimately improving patient outcomes.

In conclusion, the findings of our study provide valuable insights into a second HFx risk factor and can contribute to the development of more accurate and personalized risk assessment tools, potentially leading to the improved identification and prevention of such events. To further establish the impact of our study, one of the potential future steps would be the external validation of our model using other real-world datasets. Importantly, the present study highlights the importance of continued research with new methodologies in the field of ML, such as NLP, which may help to further refine risk prediction models for patients with a history of HFx.

### Supplementary Information

Supplementary Information.

## Data Availability

The datasets generated and analysed during the current study are not publicly available due to private commercial relationship with the study sponsor but are available from the corresponding author on reasonable request.
