# Supplementary material for: Predictive model for a second hip fracture occurrence using natural language processing and machine learning on electronic health records
Source: Sci Rep. 2024 Jan 4;14:532. doi: 10.1038/s41598-023-50762-5 (PMC10766963; doi:10.1038/s41598-023-50762-5)

Suppl. Table 1. EHRead reading performance.

|  | **TP** | **FP** | **FN** | **Predictions** | **Annotations** | **Precision** | **Recall** | **F1- score** |
| --- | --- | --- | --- | --- | --- | --- | --- | --- |
| HFx | 641 | 3 | 16 | 644 | 657 | 0.99 | 0.98 | 0.99 |
| Alcohol consumption | 27 | 1 | 0 | 28 | 27 | 0.96 | 1 | 0.98 |
| Cognitive impairment | 168 | 0 | 6 | 168 | 174 | 1 | 0.97 | 0.98 |
| Barthel Index | 26 | 0 | 1 | 26 | 27 | 1 | 0.96 | 0.98 |
| Hyperthyroidism | 29 | 0 | 3 | 29 | 32 | 1 | 0.91 | 0.95 |
| Diabetes mellitus | 134 | 15 | 3 | 149 | 137 | 0.90 | 0.98 | 0.94 |
| Smoker | 35 | 2 | 6 | 37 | 41 | 0.95 | 0.85 | 0.90 |
| Obesity | 18 | 6 | 0 | 24 | 18 | 0.75 | 1 | 0.86 |
| Hip prosthesis | 172 | 17 | 48 | 189 | 220 | 0.91 | 0.78 | 0.84 |
| Malnutrition | 1 | 0 | 2 | 1 | 3 | 1 | 0.33 | 0.5 |
| Stroke | 12 | 59 | 0 | 71 | 12 | 0.17 | 1 | 0.29 |

TP: True Positive; FP: False Positive; FN: False Negative

Suppl. Table 2. Haematology and biochemistry values at baseline

|  | | HFx  (n=1835) | | 2HFx  (n=125) | | Overall  (n=1960) |
| --- | --- | --- | --- | --- | --- | --- |
| Leukocyte count (1000/mm3) | |  | |  | |  |
| N | | 1532 (83.50%) | | 93 (74.40%) | | 1625 (82.90%) |
| Mean (SD) | | 10.54 (9.84) | | 12.50 (15.28) | | 10.65 (10.23) |
| Median (Q1-Q3) | | 9.00 (7.00, 12.00) | | 10.00 (8.00, 12.00) | | 9.16 (7.00, 12.00) |
| (min, max) | | (1, 300) | | (4 125) | | (1, 300) |
| Platelet count (1000/mm3) | |  | |  | |  |
| N | | 1545 (84.20%) | | 95 (76.00%) | | 1640 (83.70%) |
| Mean (SD) | | 237.21 (96.63) | | 242.14 (95.87) | | 237.49 (96.56) |
| Median (Q1-Q3) | | 219.00 (176.00, 284.00) | | 233.00 (173.50, 284.50) | | 219.00 (176.00, 284.00) |
| (min, max) | | (10, 1173) | | (88, 600) | | (10, 1173) |
| HbA1c, % |  | |  | |  | |
| N | 698 (38.00%) | | 36 (28.80%) | | 734 (37.40%) | |
| Mean (SD) | 6.06 (1.24) | | 6.63 (1.96) | | 6.09 (1.29) | |
| Median (Q1-Q3) | 6.00 (5.00, 6.10) | | 6.00 (5.00, 7.00) | | 6.00 (5.00, 6.30) | |
| (min, max) | (3.0, 15.0) | | (4.9, 14.1) | | (3.0, 15.0) | |
| Total cholesterol, mg/dL |  | |  | |  | |
| N | 914 (49.80%) | | 57 (45.60%) | | 971 (49.50%) | |
| Mean (SD) | 132.60 (34.25) | | 141.54 (35.70) | | 133.12 (34.38) | |
| Median (Q1-Q3) | 129.00 (109.00, 154.00) | | 137.00 (117.00, 160.00) | | 129.00 (109.50, 154.00) | |
| (min, max) | (45, 287) | | (80, 236) | | (45, 287) | |
| HDL, mg/dL |  | |  | |  | |
| N | 658 (35.90%) | | 42 (33.60%) | | 700 (35.70%) | |
| Mean (SD) | 76.62 (29.59) | | 78.71 (33.71) | | 76.74 (29.83) | |
| Median (Q1-Q3) | 72.00 (55.00, 93.00) | | 76.00 (55.00, 93.00) | | 73.00 (55.00, 93.00) | |
| (min, max) | (19, 228) | | (18, 163) | | (18, 228) | |
| LDL, mg/dL |  | |  | |  | |
| N | 95 (5.20%) | | 5 (4.00%) | | 100 (5.10%) | |
| Mean (SD) | 43.48 (14.39) | | 40.80 (3.56) | | 43.35 (14.05) | |
| Median (Q1-Q3) | 39.00 (34.00, 54.00) | | 40.00 (38.00, 44.00) | | 39.00 (34.00, 54.00) | |
| (min, max) | (20, 89) | | (37, 45) | | (20, 89) | |
| Creatinine, mg/dL |  | |  | |  | |
| N | 904 (49.30%) | | 40 (32.00%) | | 944 (48.20%) | |
| Mean (SD) | 1.33 (1.60) | | 1.27 (0.56) | | 1.32 (1.57) | |
| Median (Q1-Q3) | 1.00 (1.00, 1.00) | | 1.00 (1.00, 1.44) | | 1.00 (1.00, 1.01) | |
| (min, max) | (0.1, 43.0) | | (0.4, 3.0) | | (0.1, 43.0) | |
| ALT, U/L |  | |  | |  | |
| N | 1374 (74.90%) | | 83 (66.40%) | | 1457 (74.30%) | |
| Mean (SD) | 29.59 (47.02) | | 42.34 (98.93) | | 30.31 (51.43) | |
| Median (Q1-Q3) | 22.00 (15.00, 31.00) | | 22.00 (16.50, 34.50) | | 22.00 (15.00, 32.00) | |
| (min, max) | (1, 961) | | (7, 775) | | (1, 961) | |
| AST, U/L |  | |  | |  | |
| N | 1510 (82.30%) | | 92 (73.60%) | | 1602 (81.70%) | |
| Mean (SD) | 32.58 (60.51) | | 34.40 (89.10) | | 32.68 (62.47) | |
| Median (Q1-Q3) | 24.00 (19.00, 32.00) | | 22.50 (18.00, 28.25) | | 24.00 (19.00, 32.00) | |
| (min, max) | (6, 1449) | | (11, 866) | | (6, 1449) | |
| GGT, U/L |  | |  | |  | |
| N | 195 (10.60%) | | 8 (6.40%) | | 203 (10.40%) | |
| Mean (SD) | 109.19 (181.85) | | 63.75 (93.03) | | 107.40 (179.27) | |
| Median (Q1-Q3) | 50.00 (21.00, 106.50) | | 27.00 (16.75, 56.50) | | 50.00 (20.50, 102.50) | |
| (min, max) | (1, 1235) | | (8, 285) | | (1, 1235) | |
| CRP, mg/L |  | |  | |  | |
| N | 1346 (73.40%) | | 78 (62.40%) | | 1424 (72.70%) | |
| Mean (SD) | 56.53 (63.97) | | 36.15 (55.01) | | 55.42 (63.67) | |
| Median (Q1-Q3) | 29.00 (8.00, 89.00) | | 12.50 (4.25, 47.25) | | 27.65 (7.00, 87.00) | |
| (min, max) | (0.2, 300.0) | | (0.5, 300.0) | | (0.2, 300.0) | |
| Prealbumin, mg/dL |  | |  | |  | |
| N | 87 (4.70%) | | 4 (3.20%) | | 91 (4.60%) | |
| Mean (SD) | 13.57 (5.84) | | 15.00 (6.27) | | 13.64 (5.83) | |
| Median (Q1-Q3) | 13.00 (10.00, 16.00) | | 14.50 (11.75, 17.75) | | 13.00 (10.00, 16.00) | |
| (min, max) | (3, 32) | | (8, 23) | | (3, 32) | |
| Phosphate, mg/dL |  | |  | |  | |
| N | 1120 (61.00%) | | 49 (39.20%) | | 1169 (59.60%) | |
| Mean (SD) | 3.40 (1.07) | | 3.37 (0.91) | | 3.39 (1.06) | |
| Median (Q1-Q3) | 3.00 (3.00, 4.00) | | 3.00 (3.00, 4.00) | | 3.00 (3.00, 4.00) | |
| (min, max) | (1, 19) | | (2, 5) | | (1, 19) | |
| Calcium, mg/dL |  | |  | |  | |
| N | 1169 (63.70%) | | 57 (45.60%) | | 1226 (62.60%) | |
| Mean (SD) | 8.43 (1.06) | | 8.33 (1.16) | | 8.43 (1.07) | |
| Median (Q1-Q3) | 8.00 (8.00, 9.00) | | 8.00 (8.00, 9.00) | | 8.00 (8.00, 9.00) | |
| (min, max) | (1, 15) | | (1, 10) | | (1, 15) | |
| Vitamin D, ng/mL |  | |  | |  | |
| N | 849 (46.30%) | | 42 (33.60%) | | 891 (45.50%) | |
| Mean (SD) | 14.51 (10.24) | | 17.32 (16.93) | | 14.64 (10.65) | |
| Median (Q1-Q3) | 12.30 (7.00, 19.00) | | 12.50 (5.25, 24.75) | | 12.30 (7.00, 19.20) | |
| (min, max) | (1, 82) | | (3, 81) | | (1, 82) | |
| INR |  | |  | |  | |
| N | 1535 (83.70%) | | 94 (75.20%) | | 1629 (83.10%) | |
| Mean (SD) | 1.29 (1.05) | | 1.25 (0.79) | | 1.29 (1.04) | |
| Median (Q1-Q3) | 1.00 (1.00, 1.00) | | 1.00 (1.00, 1.00) | | 1.00 (1.00, 1.00) | |
| (min, max) | (1, 30) | | (1, 6) | | (1,30) | |
| ALT: Alanine aminotransferase; AST: Aspartate aminotransferase; CRP: C-reactive protein; FAS: Full analysis set; GGT: Gamma-glutamyl transferase; HDL: High density lipoprotein; INR: International normalized ratio; LDL: Low density lipoprotein; SD: Standard deviation; HFx: Hip fracture; 2HFx: Second hip fracture. | | | | | | |

Data extracted and analysed considering a window of (-18 months, +1 month] around index date.

Suppl. Table 3. Comorbidities of patients in the HFx subgroup at baseline

|  | HFx | | |
| --- | --- | --- | --- |
|  | Survivors  (n=1605) | Deaths  (n=230) | Overall  (n=1835) |
| Cardiovascular risk factors, n (%) | 1400(87.2%) | 219 (95.2%) | 1619 (88.2%) |
| Dyslipidemia, n (%) | 1368 (85.2%) | 219 (95.2%) | 1587 (86.5%) |
| Hypertension, n (%) | 1126 (70.2%) | 195 (84.8%) | 1321 (72.0%) |
| Anemia, n (%) | 1135 (70.7%) | 185 (80.4%) | 1320 (71.9%) |
| Osteoporosis, n (%) | 669 (41.7%) | 67 (29.1%) | 736 (40.1%) |
| Cognitive impairment, n (%) | 497 (31.0%) | 109 (47.4%) | 606 (33.0%) |
| Diabetes mellitus, n (%) | 443 (27.6%) | 84 (36.5%) | 527 (28.7%) |
| Heart failure, n (%) | 386 (24.0%) | 120 (52.2%) | 506 (27.6%) |
| Chronic Kidney disease, n (%) | 371 (23.1%) | 122 (53.0%) | 493 (26.9%) |
| Dementia, n (%) | 321 (20.0%) | 49 (21.3%) | 370 (20.2%) |
| Hyperparathyroidism, n (%) | 293 (18.3%) | 33 (14.3%) | 326 (17.8%) |
| COPD, n (%) | 189 (11.8%) | 69 (30.0%) | 258 (14.1%) |
| Anxiety, n (%) | 207 (12.9%) | 47 (20.4%) | 254 (13.8%) |
| Rheumatoid arthritis, n (%) | 188 (11.7%) | 29 (12.6%) | 217 (11.8%) |
| Ischemic cardiopathy, n (%) | 159 (9.9%) | 39 (17.0%) | 198 (10.8%) |
| Stroke, n (%) | 129 (8.0%) | 52 (22.6%) | 181 (9.9%) |
| Hip prosthesis, n (%) | 156 (9.7%) | 22 (9.6%) | 178 (9.7%) |
| Hypothyroidism, n (%) | 140 (8.7%) | 19 (8.3%) | 159 (8.7%) |
| Osteopenia, n (%) | 117 (7.3%) | 25 (10.9%) | 142 (7.7%) |
| Parkinson disease, n (%) | 67 (4.2%) | 8 (3.5%) | 75 (4.1%) |
| Asthma, n (%) | 53 (3.3%) | 10 (4.3%) | 63 (3.4%) |
| Gout, n (%) | 55 (3.4%) | 6 (2.6%) | 61 (3.3%) |
| Hyperthyroidism, n (%) | 41 (2.6%) | 8 (3.5%) | 49 (2.7%) |
| Hyperuricemia, n (%) | 30 (1.9%) | 12 (5.2%) | 42 (2.3%) |
| Knee implant, n (%) | 32 (2.0%) | 3 (1.3%) | 35 (1.9%) |
| Dyspepsia, n (%) | 23 (1.4%) | 5 (2.2%) | 28 (1.5%) |
| Transient ischemic attack, n (%) | 3 (0.2%) | 1 (0.4%) | 4 (0.2%) |
| Paroxysmal positional vertigo, n (%) | 2 (0.1%) | 0 (0.0%) | 2 (0.1%) |
| COPD: Chronic obstructive pulmonary disease; HFx: Hip fracture. | | | |

Data extracted and analysed considering a window of (-6 months, +3 months] around index date

Suppl Figure 1. Model apparent performance per number of variables in the backward selection process


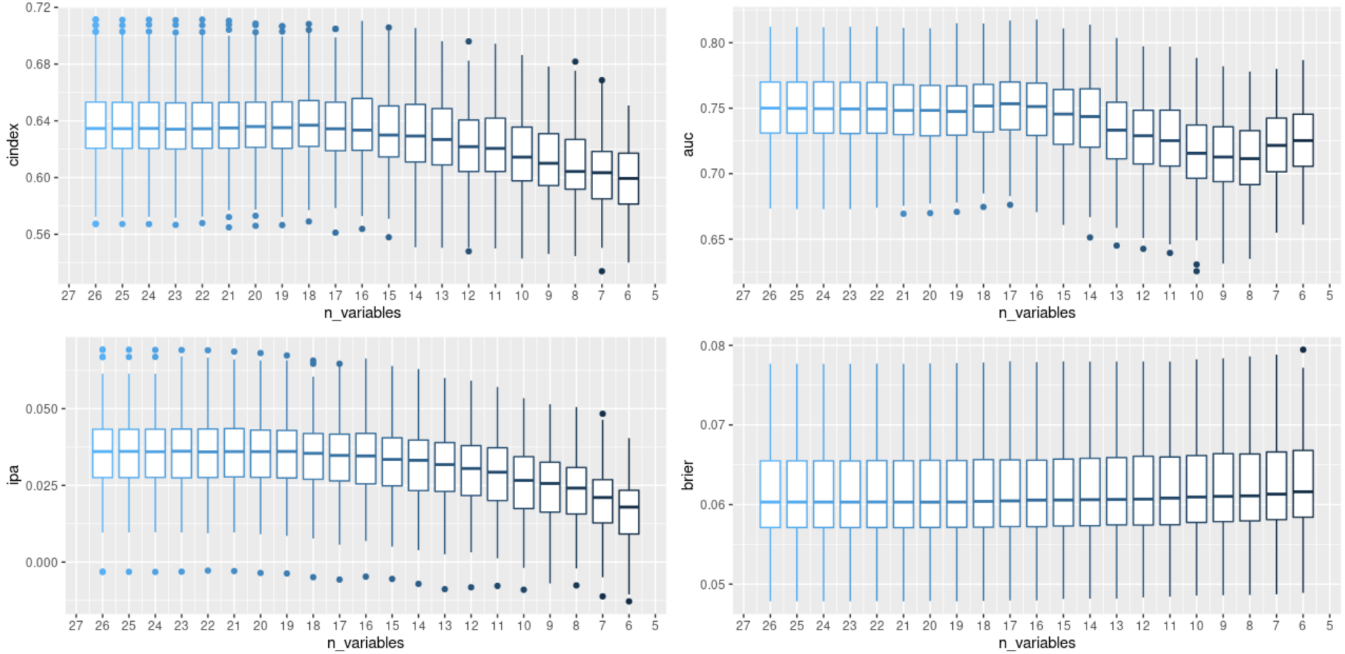

Supplement: Supplementary file 1 — Supplementary Information. [file 41598_2023_50762_MOESM1_ESM.docx]
